# Identification and Reproducibility of Urinary Metabolomic Biomarkers of Habitual Food Intake in a Cross-Sectional Analysis of the Cancer Prevention Study-3 Diet Assessment Sub-Study

**DOI:** 10.3390/metabo11040248

**Published:** 2021-04-17

**Authors:** Ying Wang, Rebecca A. Hodge, Victoria L. Stevens, Terryl J. Hartman, Marjorie L. McCullough

**Affiliations:** 1 Department of Population Science, American Cancer Society, Atlanta, GA 30303, USA; becky.hodge@cancer.org (R.A.H.); vstevens311@gmail.com (V.L.S.); marji.mccullough@cancer.org (M.L.M.); 2 Department of Epidemiology, Rollins School of Public Health, Winship Cancer Institute, Emory University, Atlanta, GA 30322, USA; terryl.johnson.hartman@emory.edu

**Keywords:** untargeted metabolomics, food biomarker, FFQ, 24 h diet recalls, urine

## Abstract

Previous cross-sectional metabolomics studies have identified many potential dietary biomarkers, mostly in blood. Few studies examined urine samples although urine is preferred for dietary biomarker discovery. Furthermore, little is known regarding the reproducibility of urinary metabolomic biomarkers over time. We aimed to identify urinary metabolomic biomarkers of diet and assess their reproducibility over time. We conducted a metabolomics analysis among 648 racially/ethnically diverse men and women in the Diet Assessment Sub-study of the Cancer Prevention Study-3 cohort to examine the correlation between >100 food groups/items [101 by a food frequency questionnaire (FFQ), and 105 by repeated 24 h diet recalls (24HRs)] and 1391 metabolites measured in 24 h urine sample replicates, six months apart. Diet–metabolite associations were examined by Pearson’s partial correlation analysis. Biomarkers were evaluated for prediction accuracy assessed using area under the curve (AUC) calculated from the receiver operating characteristic curve and for reproducibility assessed using intraclass correlation coefficients (ICCs). A total of 1708 diet–metabolite associations were identified after Bonferroni correction for multiple comparisons and restricting correlation coefficients to >0.2 or <−0.2 (1570 associations using the FFQ and 933 using 24HRs), 513 unique metabolites correlated with 79 food groups/items. The median ICCs of the 513 putative biomarkers was 0.53 (interquartile range 0.42–0.62). In this study, with comprehensive dietary data and repeated 24 h urinary metabolic profiles, we identified a large number of diet–metabolite correlations and replicated many found in previous studies. Our findings revealed the promise of urine samples for dietary biomarker discovery in a large cohort study and provide important information on biomarker reproducibility, which could facilitate their utilization in future clinical and epidemiological studies.

## 1. Introduction

Nutritional epidemiological studies have significantly advanced understanding of the relationships between diet and chronic diseases and have led to dietary guidelines for disease prevention in recent decades [1,2,3]. However, the field is still largely impeded by inconsistent findings from many studies. Most studies rely on self-reported dietary data, such as those collected from food frequency questionnaires (FFQs), which involve systematic and random measurement errors that could result in underestimated risk estimates [4]. Robust and reliable objective dietary biomarkers are important to estimate dietary intake or calibrate self-reported dietary data, thus holding promise to advancing research on diet and cancer and other health outcomes; however, such dietary biomarkers are limited to a few nutrients and do not exist for most foods and dietary patterns.

Metabolomics has shown great promise for identifying novel dietary biomarkers from human blood and urine samples in both cross-sectional and feeding studies [5]. Several large metabolomics analyses conducted in cohort studies employing a cross-sectional study design have identified hundreds of potential biomarkers of habitual food intakes [6,7,8,9,10,11,12,13] or dietary patterns [14,15]. Our previous metabolomics analyses of blood samples from the Cancer Prevention Study-II (CPS-II) Nutrition Cohort [6] and CPS-3 [13] have identified more than 200 putative food-related metabolic markers, many of which replicated findings from other population and feeding studies. The most common metabolite class was xenobiotics, which were highly correlated with plant foods. Amino acids, lipids and their metabolic end products were commonly associated with animal products such as meat, poultry, fish and dairy. Few large cohort studies have examined archived urine samples for dietary biomarkers, although urine is considered a preferred biospecimen for dietary biomarker discovery [16]. Compared with blood, urine has lower protein levels, which interfere in biomarker measurements and has a better coverage of dietary biomarkers, as more diet–metabolite associations were found in urine than in serum [11]. Furthermore, it is important to assess biomarker reproducibility to determine their future use in epidemiological and clinical studies where limited samples may be available [17]. However, little is known about urinary metabolomic biomarker reproducibility over time [18].

We previously published results on biomarkers of food intake from fasting plasma samples and their reproducibility over six months from the CPS-3 Diet Assessment Sub-study (DAS), a 12-month diet validation study [13]. In the present study, we extended our previous research to urine by utilizing the resources from the CPS-3 DAS including the post-study FFQ, repeated 24 h diet recalls (24HRs) and two 24 h urine samples collected six months apart. To fill the literature gap in the present study, we aimed to (1) identify urinary metabolites associated with individual food groups/items using untargeted metabolomics, and (2) to assess the reproducibility of identified metabolites over six months.

## 2. Results

### 2.1. Participant Characteristics

Characteristics of the study participants of the CPS-3 DAS are shown in Table 1. Among the 648 participants included in the urinary metabolomics analysis, 60.5% were white, 24.4% were black, 15.1% were Hispanic. The majority (65.0%) were female. The mean age was 52.2 ± 9.4 years.

### 2.2. 24 h Urinary Metabolites Correlated with Habitual Dietary Intake Assessed by Post-FFQ and 24HRs

We identified a total of 1708 food–metabolite associations (Supplemental Table S1), with 1570 associations using the post-FFQ (*p* < 3.56 × 10^−7^ and |r| > 0.2, Supplemental Table S2) and 933 associations using the 24HRs (*p* < 3.42 × 10^−7^ and |r| > 0.2, Supplemental Table S3); A total of 513 unique urinary metabolites were associated with 79 food groups/items assessed using either the FFQ or 24HRs, as one metabolite could be correlated with multiple food groups/items and vice versa. The majority of the diet-related metabolites were xenobiotics (*n* = 152; 29.6%), amino acids (*n* = 71, 13.8%), or unknown (*n* = 178; 34.7%); the rest were lipids (*n* = 28; 5.5%), cofactors and vitamins (*n* = 14; 2.7%), peptides (*n* = 8; 1.6%), carbohydrates (*n* = 15, 2.9%), nucleotides (*n* = 14; 2.7%) and partially characterized molecules (*n* = 27; 5.3%).

Area under the curve (AUC) of receiver operating characteristic (ROC) curve was calculated to inform how well the diet-related metabolites can discriminate top from bottom quartiles of dietary intake. The AUCs were generally higher when dietary intake was assessed using the FFQ than using 24HRs.The top 3 most predictive metabolites for each of the 79 food groups/items are shown in Table 2 (according to the post-FFQ assessment, if less than 3 metabolites are identified then top metabolites according to 24HRs were presented). The most predictive metabolite usually also had the highest |r| with a food group/item.

#### 2.2.1. Fruits

We identified 119 food–metabolite associations for 17 fruit groups/items estimated either from the FFQ or 24HRs, including 1 for grapes, 1 for prunes, 6 for bananas,19 for avocado, 2 for apples or pears, 6 for apples (24HRs only), 23 for total citrus fruits and juices, 16 for oranges, 15 for orange juice, 2 for grapefruit, 1 for watermelon, 1 for cantaloupe, 10 for berries, 3 for strawberries, 11 for blueberries, 1 for peaches and plums (Supplemental Table S1); 84 associations were observed using the FFQ (Supplemental Table S2) and 82 using the 24HRs (Supplemental Table S3). The AUCs ranged from 0.6 for vanillactate predicting prune intake assessed using the 24HRs to 0.94 for stachydrine predicting total citrus fruit and juice intake assessed by the post-FFQ.

#### 2.2.2. Vegetables

There are 150 associations for 15 vegetable groups or individual vegetables (119 associations using the FFQ, and 91 associations using the 24HRs), including 1 metabolite for ketchup and salsa, 9 for beans, 58 for all soy products, 8 for fermented soy products, 17 for soy milk, 6 for soy protein powder, 10 for cruciferous vegetables, 2 for leafy greens, 1 for iceberg or head lettuce, 2 for peppers, 7 for mushrooms, 5 for allium vegetables, 5 for onions, 17 for garlic, and 2 for garlic powder. The AUCs ranged from 0.58 for 4-acetylphenyl sulfate predicting fermented soy products assessed using the 24HRs to 0.91 for 4 metabolites predicting total bean intake assessed using the FFQ.

#### 2.2.3. Grains

We identified 35 grain–metabolite associations for total whole grains (*n* = 10), whole-grain bread (*n* = 2), whole-grain cereals (*n* = 7), corn products (*n* = 8), popcorn (*n* = 2), other whole grains (*n* = 1) and refined grains (*n* = 5); 33 associations were identified using the FFQ, and 14 using 24HRs. The AUCs ranged from 0.74 for 3,5-dihydroxybenzoic acid predicting whole-grain bread intake assessed by 24HRs to 0.91 for 2,6-dihydroxybenzoic acid predicting total whole-grain intake assessed by the FFQ.

#### 2.2.4. Proteins

We identified 404 diet–metabolite associations for 10 protein food groups/items (107 for red meat, 126 for processed meat, 119 for poultry, 7 for total fish, 6 for dark fish, 3 for shellfish, 10 for total nuts, 9 for peanuts, 9 for other nuts, and 8 for seeds); 376 associations were identified using the FFQ and 158 using 24HRs. Most metabolites correlated with red, processed meat and poultry had negative correlations with intake. The AUCs ranged from 0.69 for 3-carboxy-4-methyl-5-propyl-2-furanpropanoate (CMPF) and X-23587 predicting shellfish intake using the FFQ to 0.9 for two metabolites (tryptophan betaine and X-24412) predicting total nut intake using the FFQ.

#### 2.2.5. Dairy/Dairy Alternatives

There were 98 diet–metabolite associations for 4 dairy/dairy alternative groups (20 for milk, 4 for almond milk or rice milk, and 4 for total cheese, and 70 for cream); 93 associations were found using the FFQ, and 10 using the 24HRs. The AUCs ranged from 0.64 for X-13846 predicting almond milk or rice milk intake from 24HRs to 0.88 for heptenedioate (C7:1-DC) predicting total cheese intake from the FFQ.

#### 2.2.6. Fats and Oils

Twenty-two associations were identified for 3 fats and oils (17 for creamy salad dressing, 1 for oil and vinegar salad dressing, and 4 for olive oil); 21 were found using the FFQ and only 1 found by 24HRs. The AUCs ranged from 0.70 for 2,6-dimethylphenol sulfate predicting cream to 0.80 for *N*-methyltaurine predicting olive oil (FFQ) and oil and vinegar salad dressing (24HRs).

#### 2.2.7. Alcohol

Using either FFQ or 24HRs, we identified 443 associations for alcohol, including 136 for total alcohol, 53 for beer, 120 for wine, 104 for red wine, 24 for white wine, and 6 for liquor. 421 associations were found using the FFQ, and 243 associations were found using the 24HRs. The AUCs ranged from 0.66 for several metabolites as biomarkers of white wine intake to 0.99 for ethyl glucuronide as the biomarker of total alcohol. Ethyl glucuronide was also the most predictive metabolite for all subtypes of alcohol (beer, red wine, white wine and liquor).

#### 2.2.8. Beverages

There were 359 associations for 9 beverage groups, including 145 for total coffee, 142 for caffeinated coffee, 5 for decaffeinated coffee, 24 for total tea, 8 for green tea, 13 for black tea, 6 for herbal tea, 10 for sugar-sweetened beverages and 6 for diet beverages, with 349 found from the FFQ and 304 from 24HRs. The AUCs ranged from 0.63 for X-17686 as a biomarker of herbal tea estimated from 24HRs to 1.0 for glucuronide of C_19_H_28_O_4_ (1) and citraconate/glutaconate as biomarkers of total coffee intake. Glucuronide of C_19_H_28_O_4_ (1) is also the most predictive metabolite for caffeinated (AUC = 0.98) and decaffeinated coffee (AUC = 0.66). For tea consumption, *N*-acetyltheanine was the most predictive biomarker for total tea, green tea, and black tea but was not correlated with herbal tea intake.

#### 2.2.9. Miscellaneous

The remaining 78 associations were found for 8 miscellaneous food groups, including 22 for French fries, 20 for all chips, 12 for chocolate candies, 12 for dark chocolate, 2 for desserts, 3 for bars (breakfast, energy and high protein bars combined), 2 for soy sauce and 5 for artificial sweeteners. Acesulfame, sucralose, saccharin, erythritol and X-25785 that were associated with all artificial sweetener intake were also associated with diet beverages. The lowest AUC was 0.66 for erythritol as a biomarker of artificial sweetener intake (estimated from 24HRs); the highest AUC was 0.85 for pentose acid, abscisate for French fries (negative correlations) and X-12823 for chocolate candies (estimated from post-FFQ).

### 2.3. Reproducibility of the Identified Food Metabolites

Of the 513 metabolites that were significantly associated with food groups/items identified via FFQ or 24HRs, the median ICC for duplicate samples over six months was 0.53 (interquartile range: 0.42–0.62). By super pathway, the median ICC ranged from 0.40 for carbohydrates to 0.65 for energy metabolites.

**Figure 1:**
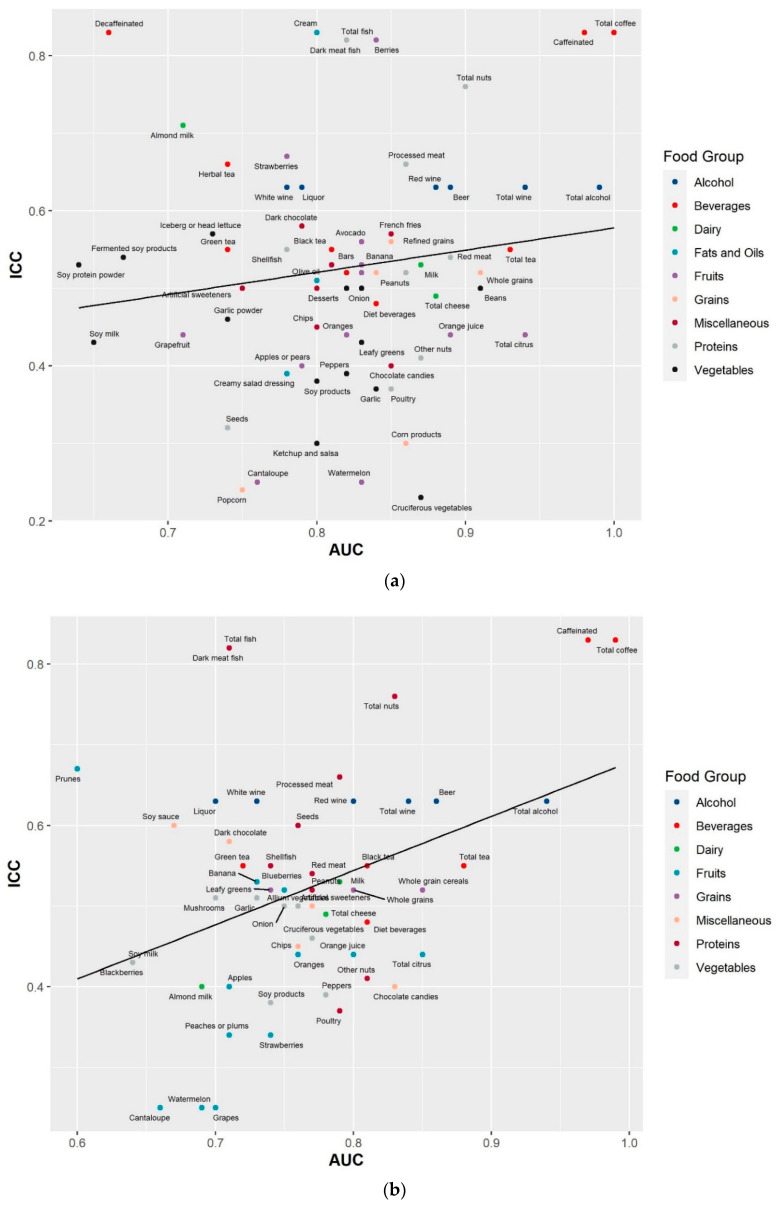
Metabolite prediction accuracy for food intake by metabolite reproducibility for the most predictive metabolite of 79 food groups/items in the Cancer Prevention Study-3 Diet Assessment Sub-study. (**a**) The most predictive metabolites for 71 food groups/items assessed using the food frequency questionnaire; (**b**) the most predictive metabolites for 60 food groups/items assessed using the average of 24 h diet recalls. Prediction accuracy was assessed by area under the curve (AUC) from the receiver operating characteristic curve, which indicates how well a metabolite could discriminate top quartile from bottom quartile intake of a food group/item. Reproducibility was assessed by intraclass correlation coefficients (ICCs), calculated as the ratio of between-person variance to the total variance among participants with repeated blood metabolic profiles measured six months apart.

**Figure 2:**
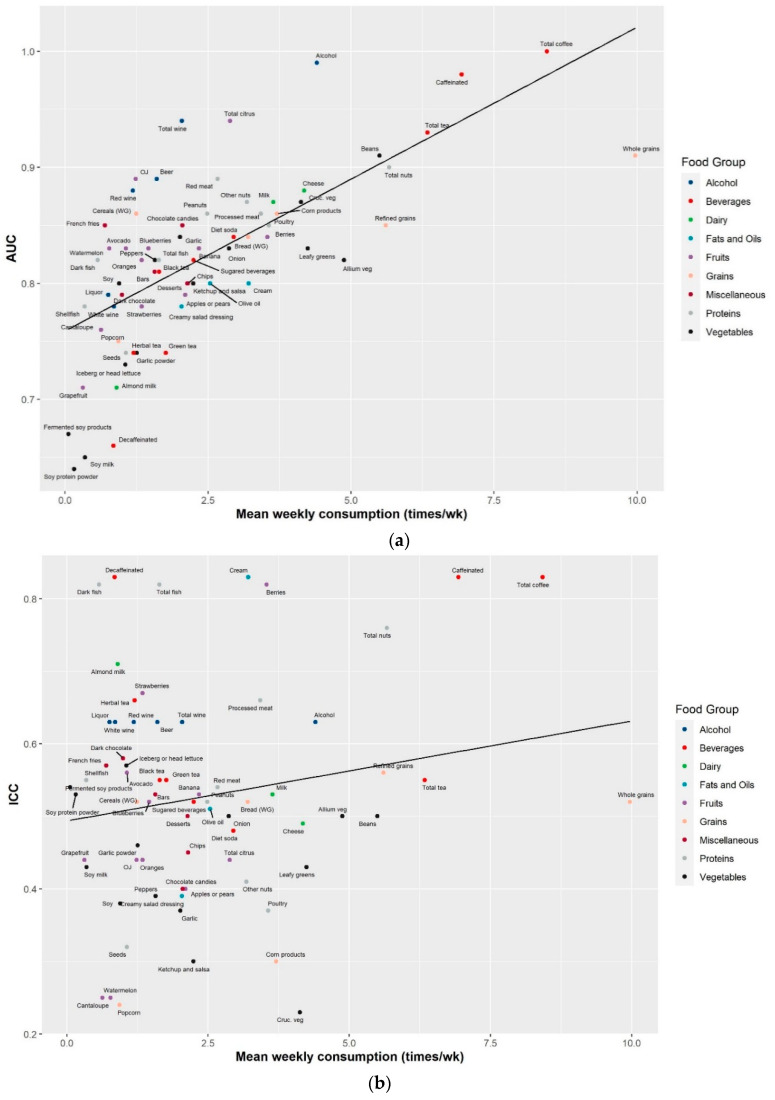
Metabolite prediction accuracy and reproducibility by food consumption frequency for the most predictive metabolite of 71 food groups/items assessed using the FFQ in the Cancer Prevention Study-3 Diet Assessment Sub-study. (**a**) Metabolite prediction accuracy, assessed by area under the curve (AUC) from the receiver operating characteristic curve, in relation to food consumption frequency; (**b**) metabolite reproducibility, assessed by intraclass correlation coefficients (ICCs) over six months, in relation to food consumption frequency.

Combining information on both prediction accuracy (AUC) and reproducibility (ICC) over time can inform the reliability of a biomarker to be used in future studies. The combined information on AUC and ICC for the most predictive metabolites of the 79 food groups/items are shown in Figure 1a,b. Biomarkers in the upper right corner with both high AUC and ICC are considered reliable, while those in the lower left corner with both low AUC and ICC are less reliable. Reliable biomarkers were seen for several food groups/items including coffee, alcohol, nuts, fish, tea, processed meat, poultry, and chocolate candies. Due to the design of DAS to capture seasonal variation by collecting 24 h urine six months apart, the low ICCs of metabolites might reflect true variation in dietary intake. We further investigated the relationship between consumption frequency in relation to AUC and ICC. Biomarkers of foods with low consumption frequencies tend to have lower AUCs and ICCs (Figure 2). Exceptions included biomarkers for fish and alcohol.

## 3. Discussion

In this cross-sectional metabolomics study among 648 men and women in the CPS-3 DAS with comprehensive dietary data assessed using both FFQ and repeated 24HRs, and with two 24 h urine samples collected approximately 6 months apart, we identified 1708 diet–metabolite correlations after adjusting for multiple comparisons. More diet–metabolite correlations were found using FFQ than 24HRs. Reproducibility of the 513 unique metabolites over six months was good for a large proportion, with 28% of metabolites with an ICC > 0.6. The comparisons of urinary dietary biomarkers identified in the present study with our previous findings in fasting plasma samples in the same study [13] revealed several overlapping food biomarkers identified in both blood and urine and many more putative biomarkers identified in urine for further evaluation. This study also provided important information on the reproducibility of the urinary biomarkers, which could facilitate their utilization in future clinical and epidemiological studies.

Urine collection is less invasive, cheaper, and offers greater volumes than blood collection. Most food components (e.g., phytochemicals) are xenobiotics that will be transformed and eliminated quickly via urine or feces. Therefore, urine as a biospecimen could be very useful for identifying dietary biomarkers in large population studies. The usefulness of urine was recently highlighted by a population study comparing dietary biomarkers measured in blood and urine samples from the same individuals. Playdon et al. [11] identified more diet–metabolite correlations in urine than in blood and more than a third of the correlations found in blood were also found in urine with similar magnitude. We previously published findings of diet-related biomarkers identified in fasting plasma samples in the CPS-3 DAS [13]. Among 671 men and women with at least one fasting blood sample in the CPS-3 DAS, a total of 677 diet–metabolite associations were identified (238 metabolites were associated with 76 food groups/items). In the present study, among a similar number of participants with at least one 24 h urine sample we identified a greater number of associations (*n* = 1708). We also found many overlapping diet–metabolite correlations in urine as we found previously in fasting plasma samples in the same study. For example, the same plausible biomarkers (food constituents or derivatives) were found for apples or pears (4-allphenol sulfate), citrus fruits and juices (stachydrine, *N*-methylhydroxyproline, *N*-methylproline), soy products (genistein glucuronide), cruciferous vegetables (*S*-methylcycteine or *S*-methylcycteine sulfoxide), garlic (alliin, *N*-acetylalliin), whole grains (2,6-dihydroxybenzoic acid, 2-acetamidophenol sulfate, 4-methoxyphenol sulfate, 2-aminophenol sulfate), poultry (3-methylhistidine), fish (CMPF), nuts (tryptophan betaine, 4-vinylphenol sulfate), milk (*N,N,N*-trimethyl-5-aminovalerate and galactonate), artificial sweeteners (acesulfame, saccharin, and erythritol), alcohol (ethyl glucuronide and ethyl α-glucopyranoside), coffee (e.g., quinate, 3-hydroxypyridine sulfate, trigonelline (*N*-methylnicotinate)), and diet beverages (acesulfame). We previously found theanine, a potentially specific biomarker of tea intake in blood [6,13]. A derivative of theanine, *N*-acetyltheanine, was found to be the most predictive biomarker of tea in urine in the present study. The magnitude of the correlations was similar in blood and urine. We also observed similar ICCs for the same biomarkers measured in both blood and urine. The high consistency between blood and urine findings in the CPS-3 DAS is also likely influenced by the fact that 24 h urine samples were returned on the same day when fasting blood samples were collected from the same participants.

We additionally replicated many other plausible biomarkers found in previous feeding or population studies, from either blood or urine. For example, we replicated biomarkers for banana (dopamine 3-O-sulfate) [6], citrus fruits and juices (e.g., *N*-methylglutamate, chiro-inositol, naringenin 7-glucuronide) [11,19], berries (catechol sulfate) [20], soy products (daidzein, genistein, daidzein sulfate, genistein sulfate, daidzein glucuronide and genistein glucuronide) [21,22], cruciferous vegetables (sulforaphane, sulforaphane-*N*-acetyl-cysteine) [23], garlic (*S*-allylcysteine, *N*-acetyl-*S* allyl-L-cysteine) [6,24,25], whole grains (3-methoxycatechol sulfate) [26], milk (phenylacetylglycine, 2,8-quinolinediol sulfate) [6], and coffee (citraconate/glutaconate, feruloylquinate, 2-Furoylglycine) [6,12,27]. There are many other potentially novel biomarkers identified in the present study which need to be confirmed in other studies and further evaluated.

Reproducibility of food-based biomarkers, affected by many sources of variability, is very important to inform the application of such biomarkers in large-scale clinical and epidemiological studies [17]. Large within-person variation in the biomarker over time is a major source of measurement errors that could lead to underestimated diet-disease risk estimates and inconsistent findings. Generally, we found lower reproducibility (or ICCs) for urinary biomarkers than for blood biomarkers, with a median ICC being 0.53 vs. 0.56 [13]. It is likely because most urinary biomarkers are xenobiotics and amino acids that are hydrophilic which have shorter half-lives than lipophilic biomarkers. Many polyphenol biomarkers have half-lives shorter than 24 h [28]. Metabolites with a short half-life tend to have a higher within-person variation, and thus a lower ICC. However, some may still be useful to capture habitual diet if the food/beverage is consumed frequently in the population (e.g., coffee), as we observed a positive relationship between consumption frequency and reproducibility of the biomarkers. Although our goal is to identify reliable biomarkers for habitual dietary intake, sensitive and specific short-term biomarkers, such as isoflavones and their derivatives for soy products, are still useful in monitoring dietary compliance in intervention studies or in populations with higher frequency of consumption. On the other hand, lipophilic or erythrocyte-associated biomarkers have longer half-lives in weeks or months because of the equilibrium of biomarkers between blood and fatty tissues, or because of binding to red blood cells [5]; thus, are useful as long-term biomarkers. For example, even though fish and alcohol were not frequently consumed among participants in the present study, their most predictive metabolites (CMPF and ethyl glucuronide, respectively) still had high reproducibility over the six-month period.

Plausible biomarkers should have positive correlations with food intake. Many metabolites were inversely correlated with foods such as red and processed meat and may not be good candidates for further evaluation. A large proportion of the diet-related metabolites are unknowns which need annotation in future studies. We reported the unknowns herein given their strong relationships with dietary factors, so they may be compared with future studies using this platform. Moving forward, more research is needed to systematically evaluate plausible food and food group biomarkers in multiple aspects such as robustness in different populations and study settings, half-lives, dose–response relationships over a range of intakes, and comparisons to benchmark biomarkers [29].

The present study has several strengths, including its large sample size, comprehensive dietary data collected using both an FFQ and repeated 24HRs, availability of 24 h urine samples, and metabolomic profile data measured by an untargeted and sensitive mass spectrometry-based approach. These rich resources enabled us to explore a large number of diet–metabolite correlations simultaneously. The repeated measures of 24 h urinary metabolic profiles make the study unique because most cohort studies did not collect urine samples or only collected spot urine and because the repeated measures allowed for an assessment of biomarker reproducibility over time. This study also has limitations. Metabolites with low correlation coefficients may not be ideal biomarkers as they only explain a small portion of the variation in dietary intake. The low correlations do not exclude them from further evaluation as candidate dietary biomarkers though, as diet was assessed using self-reported instruments in this study that have measurement errors which could attenuate the correlation estimates with biomarkers. We were not able to distinguish acute intake biomarkers from habitual dietary biomarkers as the study was designed to not to burden the participants by collection 24HRs and biospecimens at the same time. Future studies need to confirm these biomarkers in spot urine samples as 24 h urine collections are burdensome and generally not feasible in large population studies.

## 4. Materials and Methods

### 4.1. Study Population

The Diet Assessment Sub-study (DAS) was a one-year observational study among 745 men and women enrolled in the CPS-3, designed to evaluate the validity and reproducibility of the newly modified CPS-3 FFQ over a year. CPS-3 is a large prospective cohort study of 303,682 adults aged 30–65 residing in 35 states plus the District of Columbia and Puerto Rico, who were enrolled between 2006 and 2013 as described in detail elsewhere [30]. Briefly, at enrollment, participants provided a blood sample, had waist circumference measured and completed an enrollment survey. Most participants also completed a more comprehensive baseline survey that assessed extensive lifestyle, medical and other information. Follow-up questionnaires were sent in 2015 to those who completed the baseline survey after enrollment (*n* = 254,650) to update lifestyle and medical information and to assess diet using the CPS-3 FFQ for the first time.

To recruit participants to the DAS, CPS-3 participants living in 5 regions defined by Quest Diagnostics business units (Atlanta, GA, USA; Dallas, TX, USA; Auburn Hills, MI, USA; West Hills, CA, USA; San Jose, CA, USA) were invited. Enrolled participants were asked to complete the 2015 follow-up survey (to serve as the pre-FFQ), six telephone-administered 24HRs throughout the year, provide two fasting blood and two 24 h urine samples and complete a post-FFQ at the end of the study. The six 24HRs aimed to include four weekdays and two weekend days. Blood and urine samples were collected approximately six months apart to capture seasonal variation.

A total of 745 men and women met the minimum inclusion criteria of completing both pre- and post-FFQs and the first 24HR. For the urinary metabolomics analysis, we excluded participants who completed less than three 24HRs (*n* = 2), had poor post-FFQs (*n* = 20; defined as missing 2 or more sections, an entire page, >100 line items, or with daily energy intake <800 or >4500 kcal for men, and <600 or >3800 kcal for women), or had missing or invalid urine collections at both time points (*n* = 30). Invalid urine collections were defined as missed or spilled voiding ≥2 times, incorrect collection or flushing of the next morning samples, missing volume or extreme total volume (top and bottom 1% distribution), extreme urinary creatinine (top and bottom 1% distribution), or total collection period <20 or >28 h. We further excluded current smokers (*n* = 19), those whose body weight was missing at both urine collection appointments (*n* = 1) or weight change was >20 lbs between urine collections (*n* = 13), and pregnant women (*n* = 12). Finally, 648 men and women were included in the urinary metabolomics analysis (Supplemental Figure S1). Those with two eligible urine samples (*n* = 482) were included in the analysis of assessing reproducibility. The CPS-3 DAS protocol was approved by the Emory University (Atlanta, GA, USA) Institutional Review Board.

### 4.2. Diet Assessment

Diet was assessed using the newly modified CPS-3 FFQ as described elsewhere [31]. Briefly, the Willett FFQ [32,33] was modified for the CPS-3 study population, of which 17.3% were non-white participants. Modifications to the FFQ were informed through telephone-administered 24HRs, analyses of NHANES 2009-2010, and focus groups. The final modified FFQ included 191-line items. We defined 101 food groups/items from the FFQ as shown in Supplemental Table S4, generally consistent with the definitions in our previous analysis in the CPS-II Nutrition Cohort [6]. Comparable food groups were derived from the 24HRs to match those from the FFQ. We also created a few food groups using the 24HRs that are not asked (e.g., mushroom) or asked in combination with other foods (e.g., apples) on the FFQ. A total of 105 food groups/items were derived from the 24HRs. Only the post-FFQ was used in the present study as it assessed average dietary intake in the past 12 months during which period 24 h urine samples were collected.

### 4.3. 24 h Urine Collection and Processing

Participants were instructed to begin 24 h urine collections in the morning the day prior to their fasting blood collection appointment. Urine collection started after voiding the first specimen in the morning, and participants collected all urine for the next 24 h including the following morning’s first specimen. Urine was collected in 3 L unpreserved jugs, and participants were instructed to refrigerate or keep samples in a cooler with cool packs provided. The following morning, participants delivered their completed 24 h urine collection to a Quest Patient Service Center and volume was recorded. Urine specimens were then transported to a Quest Diagnostics regional processing laboratory where samples were aliquoted into 4 × 5 mL and 5 × 1.8 mL labeled cryovials. All aliquots were frozen and shipped on dry ice to an off-site biorepository (Fisher BioServices, Inc., Frederick, MD, USA) for long-term storage in the vapor phase of liquid nitrogen.

### 4.4. Metabolomics Analysis

Metabolomic profiling was conducted by Metabolon, Inc. (Durham, NC, USA) using ultrahigh performance liquid chromatography-tandem mass spectrometry (UPLC–MS/MS) described in detail elsewhere [34,35]. Briefly, 100 µL urine samples were treated with 450 µL of methanol to precipitate proteins using an automated liquid handling robot (Hamilton LabStar, Hamilton Robotics, Inc., Reno, NV, USA). Four sample fractions were dried and reconstituted in different solvents for measurement under four different platforms. Two aliquots were analyzed using two separate reverse phase (RP)/UPLC–MS/MS methods with positive ion mode electrospray ionization (ESI), one chromatographically optimized for more hydrophilic compounds and one for more hydrophobic compounds. Another aliquot was analyzed using RP/UPLC–MS/MS with negative ion mode ESI using a separate dedicated C18 column. The last aliquot was analyzed via hydrophilic interaction chromatography (HILIC)/UPLC–MS/MS with negative ion mode ESI. Mobile phases of the RP positive ion method consisted of 0.1% formic acid in water and 0.1% formic acid in methanol. Mobile phases of the RP negative ion method consisted of 6.5 mM ammonium bicarbonate in water (pH 8) and 6.5 mM ammonium bicarbonate in 95% methanol/5% water. Mobile phases of the HILIC method consisted of 10 mM ammonium formate in 15% water, 5% methanol, 80% acetonitrile and 10 mM ammonium formate in 50% water, 50% acetonitrile. For all methods, the injection volume was 5 µL and a 2× needle loop overfill was used. Individual metabolites were identified by comparison with a chemical library maintained by Metabolon that comprises more than 3300 authenticated standards and recurrent unknown entities, based on retention time/index, mass to charge ratio, and chromatographic data (including MS/MS spectral data).

A total of 1551 metabolites were detected in the 24 h urine samples. Metabolites that were below the detection limit in >90% of the samples were excluded (*n* = 147). Values for each sample were normalized by osmolality. To correct the day-to-day variation from the platform, each metabolite was then rescaled to set the median equal to 1. Lastly, missing values are imputed with the minimum. Triplicates of 44 participant samples were used as quality controls to assess inter- and intra-batch variation. Intraclass correlation coefficients (ICCs) were calculated among the quality control samples to test the reproducibility of the platforms. Metabolites with an ICC < 0.5 were further excluded from the analysis, leaving 1391 for diet–metabolite analysis. Of the 1391 included metabolites, the median technical ICC was 0.94 (interquartile range: 0.89 to 0.97), suggesting a very high reproducibility of the platforms.

### 4.5. Statistical Analysis

Metabolite and food variables (from FFQs and 24HRs) were generalized log transformed [36] and auto-scaled before all analyses. Metabolite levels were averaged for participants with two measurements. Pearson’s partial correlation was used to determine the food–metabolite correlations, controlling for age (continuous), gender, race/ethnicity (white, black, Hispanic), education (no college, college graduate, graduate school, unknown), smoking status (never, former), physical activity (metabolic equivalent hours per week (MET-h/wk): <5, 5–<10 or missing, 10–<15, ≥15), body mass index (kg/m^2^, continuous), ethanol intake (g/d, continuous; except for alcohol-containing items), and energy intake (kcal/d, continuous). Associations were considered statistically significant if *p* values were less than the Bonferroni-corrected threshold (0.05/1391/101 = 3.56 × 10^−7^ for FFQ, 0.05/1391/105 = 3.42 × 10^−7^ for 24HRs). To minimize false-positive findings, we further required the absolute values of the correlation coefficient (|r|) were greater than 0.2.

Putative dietary biomarkers were further evaluated for predictive accuracy of discriminating top from bottom quartile of consumption (highest vs. lowest intake), assessed using the AUC calculated from the ROC curve using R package pROC [37]. AUC < 0.7 was considered to be low, 0.7–<0.8 to be moderate, and ≥0.8 to be high.

The reproducibility of the identified food-related metabolites over six months was assessed using ICCs. ICCs were calculated as the ratio of between-person variance to the total variance among participants with repeated measures of urinary metabolic profiles. Between-person variance was estimated from a random effects model where participants were modeled as a random variable. We considered ICCs > 0.6 to be good and >0.75 to be excellent reproducibility.

## 5. Conclusions

In conclusion, in this large cross-sectional analysis of habitual diet and 24 h urinary metabolic profiles in a free-living population of 648 racially/ethnically diverse men and women, we identified many more potential dietary biomarkers in urine than fasting blood samples in the same study, and replicated several found in other previous studies. These findings provided complimentary information to blood biomarkers and important information on the reproducibility of the urinary biomarkers. These candidate biomarkers warrant further evaluation and reliable ones could be used in future clinical and epidemiological studies.

## Figures and Tables

**Table 1:** Characteristics of participants (*n* = 648) in the Cancer Prevention Study-3 Diet Assessment Sub-study ^1^.

Characteristics	Men (*n* = 227)	Women (*n* = 421)
Age (year)	52.3 ± 10.1	52.2 ± 9.1
Race/ethnicity		
White	146 (64.3)	246 (58.4)
Black	38 (16.7)	120 (28.5)
Hispanic	43 (18.9)	55 (13.1)
BMI at pre-FFQ (kg/m^2^)	27.2 (5.0)	27.8 (6.5)
Education		
<College	39 (17.2)	104 (24.7)
College	78 (34.4)	137 (32.5)
≥Graduate school	101 (44.5)	167 (39.7)
Unknown	9 (4.0)	13 (3.1)
Smoking status		
Never	178 (78.4)	336 (79.8)
Former	49 (21.6)	85 (20.2)
Recreational physical activity (MET-h/wk)		
0–<5	41 (18.1)	120 (28.5)
5–<10 ^2^	71 (31.3)	143 (34.0)
10–<15	49 (21.6)	72 (17.1)
≥15	66 (29.1)	86 (20.4)
Ethanol intake (g/d)	10.4 ± 14.1	6.8 ± 11.1
Energy from post-FFQ (kcal/d)	2134 ± 687	2001 ± 611
Average energy intake from 24HRs (kcal/d)	2198 ± 570	1724 ± 407

Abbreviations: BMI, body mass index; 24HR, 24 h diet recall; FFQ, food frequency questionnaire; MET-h, metabolic equivalent hour. ^1^ Values are the mean ± standard deviation for continuous variables, and frequency (%) for categorical variables. ^2^ Includes missing.

**Table 2:** Top three predictive metabolites for 79 food group/item assessed using the CPS-3 FFQ and average of 24 h diet recalls in the Cancer Prevention Study-3 Diet Assessment Sub-study ^1^.

Food Group/Items	Biochemical Name ^2^	Super Pathway	Sub Pathway	Post-FFQ			Average 24HRs			ICC ^3^
				R	*p* Value	AUC	R	*p* Value	AUC	
**FRUITS**										
Grapes	naringenin 7-glucuronide	Xenobiotics	Food Component/Plant	0.10	1.52 × 10^−2^	0.73	**0.21**	**1.42 × 10^−7^**	0.70	0.25 (0.17, 0.34)
Prunes	vanillactate	Amino Acid	Tyrosine Metabolism	0.11	7.29 × 10^−3^	0.63	**0.21**	**1.68 × 10^−7^**	0.60	0.67 (0.62, 0.72)
Banana	dopamine 3-*O*-sulfate	Amino Acid	Tyrosine Metabolism	**0.30**	**6.60 × 10^−15^**	0.83	**0.25**	**1.48 × 10^−10^**	0.73	0.53 (0.47, 0.60)
	X-24338			**0.30**	**3.37 × 10^−14^**	0.82	**0.27**	**5.94 × 10^−12^**	0.73	0.40 (0.33, 0.48)
	ethyl pyruvate	Xenobiotics	Food Component/Plant	**0.25**	**2.24 × 10^−10^**	0.80	**0.25**	**1.17 × 10^−10^**	0.74	0.38 (0.31, 0.46)
Avocado	3-methyladipate	Lipid	Fatty Acid, Dicarboxylate	**0.27**	**2.91 × 10^−12^**	0.83	0.18	7.13 × 10^−6^	0.74	0.56 (0.50, 0.62)
	homocitrate	Xenobiotics	Food Component/Plant	**0.21**	**1.75 × 10^−7^**	0.82	0.13	8.34 × 10^−4^	0.74	0.50 (0.44, 0.57)
	X-17335			**0.21**	**1.23 × 10^−7^**	0.82	0.08	5.29 × 10^−2^	0.72	0.38 (0.31, 0.46)
Apples or pears	4-allylphenol sulfate	Xenobiotics	Food Component/Plant	**0.21**	**1.34 × 10^−7^**	0.79				0.40 (0.32, 0.47)
	xylose	Carbohydrate	Pentose Metabolism	**0.21**	**1.74 × 10^−7^**	0.77				0.34 (0.27, 0.43)
Apples ^4^	4-allylphenol sulfate	Xenobiotics	Food Component/Plant				**0.25**	**1.10 × 10^−10^**	0.71	0.40 (0.32, 0.47)
	xylose	Carbohydrate	Pentose Metabolism				**0.24**	**1.94 × 10^−9^**	0.71	0.34 (0.27, 0.43)
	X-25838						**0.25**	**2.01 × 10^−10^**	0.70	0.40 (0.32, 0.47)
Total citrus fruits and juices	stachydrine	Xenobiotics	Food Component/Plant	**0.52**	**7.41 × 10^−45^**	0.94	**0.46**	**6.06 × 10^−35^**	0.85	0.44 (0.37, 0.51)
	*N*-methylglutamate	Amino Acid	Glutamate Metabolism	**0.46**	**4.00 × 10^−34^**	0.90	**0.39**	**1.90 × 10^−24^**	0.82	0.47 (0.40, 0.54)
	X-12111			**0.40**	**4.24 × 10^−26^**	0.90	**0.40**	**9.72 × 10^−26^**	0.82	0.40 (0.33, 0.48)
Oranges	stachydrine	Xenobiotics	Food Component/Plant	**0.30**	**5.24 × 10^−15^**	0.82	**0.27**	**5.55 × 10^−12^**	0.76	0.44 (0.37, 0.51)
	*N*-methylglutamate	Amino Acid	Glutamate Metabolism	**0.25**	**1.23 × 10^−10^**	0.81	**0.20**	**2.09 × 10^−7^**	0.73	0.47 (0.40, 0.54)
	X-19183			**0.24**	**1.94 × 10^−9^**	0.79	**0.25**	**1.42 × 10^−10^**	0.75	0.34 (0.27, 0.42)
Orange juice	stachydrine	Xenobiotics	Food Component/Plant	**0.36**	**9.49 × 10^−21^**	0.89	**0.35**	**9.29 × 10^−20^**	0.80	0.44 (0.37, 0.51)
	*N*-methylglutamate	Amino Acid	Glutamate Metabolism	**0.36**	**2.62 × 10^−20^**	0.88	**0.34**	**2.14 × 10^−18^**	0.79	0.47 (0.40, 0.54)
	X-12111			**0.32**	**1.35 × 10^−16^**	0.87	**0.35**	**1.38 × 10^−19^**	0.79	0.40 (0.33, 0.48)
Grapefruit	stachydrine	Xenobiotics	Food Component/Plant	**0.25**	**1.87 × 10^−10^**	0.71	0.18	7.47 × 10^−6^	0.62	0.44 (0.37, 0.51)
	*N*-methylglutamate	Amino Acid	Glutamate Metabolism	**0.21**	**8.50 × 10^−8^**	0.71	0.15	1.22 × 10^−4^	0.61	0.47 (0.40, 0.54)
Watermelon	X-25271			**0.38**	**1.43 × 10^−23^**	0.83	**0.31**	**6.53 × 10^−16^**	0.69	0.25 (0.17, 0.34)
Cantaloupe	X-25271			**0.31**	**2.17 × 10^−15^**	0.76	**0.21**	**1.88 × 10^−7^**	0.66	0.25 (0.17, 0.34)
Berries	quinate	Xenobiotics	Food Component/Plant	**0.21**	**5.87 × 10^−8^**	0.84	0.10	1.54 × 10^−2^	0.71	0.82 (0.79, 0.85)
	4-allylphenol sulfate	Xenobiotics	Food Component/Plant	**0.20**	**2.67 × 10^−7^**	0.83	0.19	2.05 × 10^−6^	0.75	0.40 (0.32, 0.47)
	X-24757			**0.23**	**4.37 × 10^−9^**	0.82	0.12	3.62 × 10^−3^	0.72	0.64 (0.59, 0.69)
Strawberries	xylose	Carbohydrate	Pentose Metabolism	0.19	8.56 × 10^−7^	0.78	**0.22**	**3.30 × 10^−8^**	0.74	0.34 (0.27, 0.43)
	X-25523			0.15	1.56 × 10^−4^	0.78	**0.21**	**7.47 × 10^−8^**	0.72	0.49 (0.42, 0.56)
	ursocholate	Lipid	Secondary Bile Acid Metabolism	**−0.21**	**1.12 × 10^−7^**	0.78	−0.06	1.58 × 10^−1^	0.69	0.67 (0.61, 0.71)
Blueberries	X-23970			**0.22**	**1.26 × 10^−8^**	0.83	**0.24**	**4.83 × 10^−10^**	0.75	0.52 (0.46, 0.59)
	X-25523			**0.22**	**2.00 × 10^−8^**	0.83	**0.21**	**1.09 × 10^−7^**	0.73	0.49 (0.42, 0.56)
	catechol sulfate	Xenobiotics	Benzoate Metabolism	**0.22**	**2.65 × 10^−8^**	0.83	0.15	1.50 × 10^−4^	0.72	0.71 (0.66, 0.75)
Blackberries ^4^	isocitric lactone	Energy	TCA Cycle				**0.22**	**3.49 × 10^−8^**	0.64	0.43 (0.36, 0.51)
Peaches or plums	xylose	Carbohydrate	Pentose Metabolism	0.09	2.16 × 10^−2^	0.76	**0.22**	**3.62 × 10^−8^**	0.71	0.34 (0.27, 0.43)
**VEGETABLES**										
Ketchup and salsa	X-25247			**0.21**	**1.25 × 10^−7^**	0.80	0.12	1.79 × 10^−3^	0.72	0.30 (0.23, 0.39)
Beans	X-17365			**0.23**	**7.88 × 10^−9^**	0.91	0.18	4.68 × 10^−6^	0.71	0.50 (0.43, 0.56)
	*N*-acetylalliin	Xenobiotics	Food Component/Plant	**0.23**	**2.64 × 10^−9^**	0.91	0.17	2.73 × 10^−5^	0.71	0.37 (0.30, 0.45)
	X-23639			**0.22**	**2.43 × 10^−8^**	0.91	0.12	1.72 × 10^−3^	0.70	0.66 (0.61, 0.71)
Soy products	glycitein glucuronide (2) *	Xenobiotics	Food Component/Plant	**0.39**	**6.26 × 10^−25^**	0.80	**0.39**	**5.00 × 10^−25^**	0.74	0.38 (0.31, 0.46)
	glycitein sulfate (2)	Xenobiotics	Food Component/Plant	**0.35**	**1.52 × 10^−19^**	0.79	**0.39**	**7.49 × 10^−24^**	0.75	0.46 (0.39, 0.53)
	daidzein sulfate (2)	Xenobiotics	Food Component/Plant	**0.35**	**6.36 × 10^−20^**	0.79	**0.35**	**1.54 × 10^−19^**	0.74	0.44 (0.37, 0.52)
Fermented soy products	carnosine	Amino Acid	Histidine Metabolism	**−0.20**	**2.17 × 10^−7^**	0.68	−0.10	1.01 × 10^−2^	0.60	0.40 (0.33, 0.47)
	isovalerylcarnitine (C5)	Amino Acid	Leucine, Isoleucine and Valine Metabolism	**−0.24**	**6.68 × 10^−10^**	0.67	−0.12	2.86 × 10^−3^	0.59	0.54 (0.47, 0.60)
	*N*,*N*,*N*-trimethyl-5-aminovalerate	Amino Acid	Lysine Metabolism	**−0.20**	**3.51 × 10^−7^**	0.67	−0.08	5.80 × 10^−2^	0.59	0.40 (0.33, 0.48)
Soy milk	daidzein sulfate (1)	Xenobiotics	Food Component/Plant	**0.31**	**1.97 × 10^−15^**	0.65	**0.37**	**1.85 × 10^−21^**	0.64	0.43 (0.36, 0.50)
	X-18750			**0.28**	**7.48 × 10^−13^**	0.65	**0.31**	**6.07 × 10^−16^**	0.62	0.40 (0.33, 0.47)
	glycitein sulfate (2)	Xenobiotics	Food Component/Plant	**0.30**	**9.22 × 10^−15^**	0.65	**0.31**	**5.51 × 10^−16^**	0.62	0.46 (0.39, 0.53)
Soy protein powder	X-16649			**0.21**	**9.86 × 10^−8^**	0.64	0.12	1.70 × 10^−3^	0.60	0.53 (0.46, 0.59)
	daidzein sulfate (1)	Xenobiotics	Food Component/Plant	**0.20**	**2.28 × 10^−7^**	0.64	0.10	1.16 × 10^−2^	0.60	0.43 (0.36, 0.50)
	genistein	Xenobiotics	Food Component/Plant	**0.20**	**2.68 × 10^−7^**	0.63	0.07	5.94 × 10^−2^	0.61	0.36 (0.28, 0.44)
Cruciferous vegetables	X-25217			**0.37**	**8.67 × 10^−22^**	0.87	0.19	8.94 × 10^−7^	0.74	0.23 (0.16, 0.33)
	*S*-methylcysteine sulfoxide	Amino Acid	Methionine, Cysteine, SAM and Taurine Metabolism	**0.30**	**8.50 × 10^−15^**	0.86	**0.21**	**1.50 × 10^−7^**	0.77	0.46 (0.39, 0.53)
	X-24330			**0.25**	**1.84 × 10^−10^**	0.85	0.14	3.83 × 10^−4^	0.70	0.57 (0.51, 0.63)
Leafy greens	cytosine	Nucleotide	Pyrimidine Metabolism, Cytidine containing	**0.22**	**2.39 × 10^−8^**	0.83	0.08	4.02 × 10^−2^	0.69	0.43 (0.36, 0.50)
	X-23970			0.19	1.07 × 10^−6^	0.81	**0.21**	**1.35 × 10^−7^**	0.74	0.52 (0.46, 0.59)
Iceberg or head lettuce	pentose acid *	Partially Characterized Molecules	Partially Characterized Molecules	**−0.22**	**3.45 × 10^−8^**	0.73	−0.11	4.15 × 10^−3^	0.58	0.57 (0.50, 0.62)
Peppers	X-23780			**0.28**	**9.82 × 10^−13^**	0.82	**0.21**	**8.85 × 10^−8^**	0.78	0.39 (0.31, 0.47)
	X-17365			**0.22**	**2.35 × 10^−8^**	0.80	0.16	8.55 × 10^−5^	0.75	0.50 (0.43, 0.56)
Mushrooms ^4^	*N*-methyltaurine	Amino Acid	Methionine, Cysteine, SAM and Taurine Metabolism				**0.23**	**4.77 × 10^−9^**	0.70	0.51 (0.44, 0.57)
	X-17365						**0.22**	**4.53 × 10^−8^**	0.70	0.50 (0.43, 0.56)
	*N*-acetylalliin	Xenobiotics	Food Component/Plant				**0.21**	**9.37 × 10^−8^**	0.70	0.37 (0.30, 0.45)
Allium vegetables	X-17365			**0.37**	**4.08 × 10^−22^**	0.82	**0.22**	**3.19 × 10^−8^**	0.76	0.50 (0.43, 0.56)
	*N*-methyltaurine	Amino Acid	Methionine, Cysteine, SAM and Taurine Metabolism	**0.34**	**2.14 × 10^−18^**	0.82	**0.24**	**7.67 × 10^−10^**	0.75	0.51 (0.44, 0.57)
	2,3-dimethylsuccinate	Amino Acid	Leucine, Isoleucine and Valine Metabolism	**0.29**	**1.79 × 10^−13^**	0.81	**0.20**	**2.10 × 10^−7^**	0.74	0.36 (0.28, 0.44)
Onion	X-17365			**0.36**	**1.10 × 10^−20^**	0.83	**0.21**	**9.24 × 10^−8^**	0.75	0.50 (0.43, 0.56)
	*N*-methyltaurine	Amino Acid	Methionine, Cysteine, SAM and Taurine Metabolism	**0.33**	**2.65 × 10^−17^**	0.83	**0.23**	**2.79 × 10^−9^**	0.73	0.51 (0.44, 0.57)
	2,3-dimethylsuccinate	Amino Acid	Leucine, Isoleucine and Valine Metabolism	**0.28**	**1.12 × 10^−12^**	0.81	0.20	4.48 × 10^−7^	0.72	0.36 (0.28, 0.44)
Garlic	*N*-acetylalliin	Xenobiotics	Food Component/Plant	**0.38**	**2.58 × 10^−23^**	0.84	**0.20**	**3.59 × 10^−7^**	0.71	0.37 (0.30, 0.45)
	*N*-methyltaurine	Amino Acid	Methionine, Cysteine, SAM and Taurine Metabolism	**0.23**	**9.41 × 10^−9^**	0.82	**0.21**	**8.04 × 10^−8^**	0.73	0.51 (0.44, 0.57)
	X-17365			**0.29**	**5.77 × 10^−14^**	0.82	**0.21**	**1.49 × 10^−7^**	0.72	0.50 (0.43, 0.56)
Garlic powder	*N*-acetyl-*S*-allyl-L-cysteine	Xenobiotics	Food Component/Plant	**0.23**	**5.31 × 10^−9^**	0.74	0.04	2.72 × 10^−1^	0.69	0.46 (0.39, 0.53)
	*S*-allylcysteine	Xenobiotics	Food Component/Plant	**0.22**	**2.18 × 10^−8^**	0.73	0.06	1.07 × 10^−1^	0.69	0.35 (0.28, 0.44)
**GRAINS**										
Whole grains	2,6-dihydroxybenzoic acid	Xenobiotics	Drug-Topical Agents	**0.32**	**5.70 × 10^−17^**	0.91	**0.23**	**2.50 × 10^−9^**	0.80	0.52 (0.45, 0.58)
	2-acetamidophenol sulfate	Xenobiotics	Drug-Analgesics, Anesthetics	**0.34**	**3.29 × 10^−18^**	0.90	**0.26**	**5.93 × 10^−11^**	0.80	0.51 (0.44, 0.57)
	4-methoxyphenol sulfate	Amino Acid	Tyrosine Metabolism	**0.27**	**7.24 × 10^−12^**	0.89	0.16	8.07 × 10^−5^	0.77	0.33 (0.26, 0.41)
Whole-grain bread	3,5-dihydroxybenzoic acid	Xenobiotics	Food Component/Plant	**0.31**	**1.42 × 10^−15^**	0.84	**0.28**	**1.53 × 10^−12^**	0.74	0.52 (0.46, 0.59)
	2-acetamidophenol sulfate	Xenobiotics	Drug-Analgesics, Anesthetics	**0.23**	**2.99 × 10^−9^**	0.81	0.19	1.67 × 10^−6^	0.71	0.51 (0.44, 0.57)
Whole-grain cereals	2,6-dihydroxybenzoic acid	Xenobiotics	Drug-Topical Agents	**0.36**	**2.30 × 10^−20^**	0.86	**0.35**	**1.62 × 10^−19^**	0.85	0.52 (0.45, 0.58)
	2-acetamidophenol sulfate	Xenobiotics	Drug-Analgesics, Anesthetics	**0.36**	**1.07 × 10^−20^**	0.85	**0.31**	**2.85 × 10^−15^**	0.82	0.51 (0.44, 0.57)
	2-aminophenol sulfate	Xenobiotics	Chemical	**0.30**	**6.27 × 10^−15^**	0.83	**0.28**	**1.13 × 10^−12^**	0.80	0.45 (0.39, 0.53)
Corn products	X-25247			**0.32**	**1.19 × 10^−16^**	0.86	0.12	1.93 × 10^−3^	0.73	0.30 (0.23, 0.39)
	X-23680			**0.26**	**2.38 × 10^−11^**	0.86	0.11	5.39 × 10^−3^	0.73	0.56 (0.50, 0.62)
	carnitine of C_10_H_14_O_2_ (2) *	Partially Characterized Molecules	Partially Characterized Molecules	**0.21**	**5.12 × 10^−8^**	0.85	0.05	1.95 × 10^−1^	0.72	0.39 (0.32, 0.47)
Popcorn	glucuronide of C_12_H_20_O_3_ (1) *	Partially Characterized Molecules	Partially Characterized Molecules	**0.24**	**6.28 × 10^−10^**	0.75	0.19	8.12 × 10^−7^	0.70	0.24 (0.16, 0.33)
	X-25247			**0.26**	**6.63 × 10^−11^**	0.75	0.16	4.24 × 10^−5^	0.69	0.30 (0.23, 0.39)
Other whole grains	3,5-dihydroxybenzoic acid	Xenobiotics	Food Component/Plant	**0.20**	**2.33 × 10^−7^**	0.78	0.18	6.67 × 10^−6^	0.69	0.52 (0.46, 0.59)
Refined grains	X-23680			**0.21**	**1.78 × 10^−7^**	0.85	0.11	3.85 × 10^−3^	0.84	0.56 (0.50, 0.62)
	1,5-anhydroglucitol (1,5-AG)	Carbohydrate	Glycolysis, Gluconeogenesis, and Pyruvate Metabolism	**0.21**	**7.39 × 10^−8^**	0.83	0.08	5.81 × 10^−2^	0.84	0.42 (0.35, 0.49)
	N6-carbamoylthreonyladenosine	Nucleotide	Purine Metabolism, Adenine containing	**0.21**	**1.86 × 10^−7^**	0.83	0.01	7.49 × 10^−1^	0.83	0.64 (0.58, 0.69)
**PROTEINS**										
Red meat	isovalerylcarnitine (C5)	Amino Acid	Leucine, Isoleucine and Valine Metabolism	**0.31**	**8.67 × 10^−16^**	0.89	**0.25**	**2.07 × 10^−10^**	0.77	0.54 (0.47, 0.60)
	3,4-dihydroxyphenylacetate sulfate	Amino Acid	Tyrosine Metabolism	**−0.28**	**5.22 × 10^−13^**	0.88	**−0.26**	**6.04 × 10^−11^**	0.79	0.54 (0.48, 0.61)
	*N,N,N*-trimethyl-5-aminovalerate	Amino Acid	Lysine Metabolism	**0.31**	**9.55 × 10^−16^**	0.88	**0.23**	**5.08 × 10^−9^**	0.77	0.40 (0.33, 0.48)
Processed meat	1-ribosyl-imidazoleacetate *	Amino Acid	Histidine Metabolism	**−0.34**	**8.25 × 10^−19^**	0.86	**−0.26**	**4.02 × 10^−11^**	0.79	0.66 (0.60, 0.71)
	X-23970			**−0.31**	**3.37 × 10^−15^**	0.86	−0.16	3.16 × 10^−5^	0.78	0.52 (0.46, 0.59)
	pentose acid *	Partially Characterized Molecules	Partially Characterized Molecules	**−0.31**	**2.70 × 10^−15^**	0.85	−0.18	6.15 × 10^−6^	0.78	0.57 (0.50, 0.62)
Poultry	anserine	Amino Acid	Histidine Metabolism	**0.52**	**3.02 × 10^−44^**	0.85	**0.37**	**2.40 × 10^−22^**	0.79	0.37 (0.30, 0.45)
	3-methylhistidine	Amino Acid	Histidine Metabolism	**0.56**	**1.01 × 10^−54^**	0.84	**0.45**	**2.89 × 10^−32^**	0.82	0.46 (0.39, 0.53)
	X-13835			**0.56**	**3.71 × 10^−53^**	0.84	**0.43**	**1.67 × 10^−29^**	0.82	0.60 (0.54, 0.66)
Total fish	CMPF	Lipid	Fatty Acid, Dicarboxylate	**0.39**	**5.89 × 10^−24^**	0.82	**0.28**	**1.25 × 10^−12^**	0.71	0.82 (0.79, 0.85)
	X-25419			**0.31**	**1.96 × 10^−15^**	0.80	**0.24**	**2.02 × 10^−9^**	0.71	0.55 (0.49, 0.61)
	X-13835			**0.31**	**2.68 × 10^−15^**	0.77	0.17	2.64 × 10^−5^	0.66	0.60 (0.54, 0.66)
Dark meat fish	CMPF	Lipid	Fatty Acid, Dicarboxylate	**0.38**	**2.03 × 10^−23^**	0.82	**0.23**	**2.64 × 10^−9^**	0.71	0.82 (0.79, 0.85)
	X-25419			**0.29**	**3.68 × 10^−14^**	0.78	0.17	1.28 × 10^−5^	0.73	0.55 (0.49, 0.61)
	X-13835			**0.24**	**1.51 × 10^−9^**	0.77	0.15	1.48 × 10^−4^	0.68	0.60 (0.54, 0.66)
Shellfish	X-25419			**0.39**	**1.92 × 10^−24^**	0.78	**0.27**	**6.23 × 10^−12^**	0.74	0.55 (0.49, 0.61)
	CMPF	Lipid	Fatty Acid, Dicarboxylate	**0.24**	**1.43 × 10^−9^**	0.69	0.17	2.08 × 10^−5^	0.70	0.82 (0.79, 0.85)
	X-23587			**0.23**	**4.70 × 10^−9^**	0.69	0.13	9.62 × 10^−4^	0.68	0.56 (0.49, 0.62)
Total nuts	tryptophan betaine	Amino Acid	Tryptophan Metabolism	**0.42**	**2.59 × 10^−28^**	0.90	**0.31**	**2.38 × 10^−15^**	0.83	0.76 (0.72, 0.79)
	X-24412			**0.38**	**8.07 × 10^−24^**	0.90	**0.32**	**5.62 × 10^−17^**	0.83	0.52 (0.46, 0.59)
	X-23644			**0.31**	**7.22 × 10^−16^**	0.89	**0.26**	**3.59 × 10^−11^**	0.80	0.31 (0.24, 0.40)
Peanuts	X-24412			**0.43**	**6.25 × 10^−30^**	0.86	**0.38**	**1.01 × 10^−22^**	0.77	0.52 (0.46, 0.59)
	tryptophan betaine	Amino Acid	Tryptophan Metabolism	**0.42**	**4.80 × 10^−28^**	0.86	**0.34**	**1.98 × 10^−18^**	0.76	0.76 (0.72, 0.79)
	4-vinylphenol sulfate	Xenobiotics	Benzoate Metabolism	**0.40**	**2.88 × 10^−25^**	0.86	**0.25**	**3.70 × 10^−10^**	0.71	0.41 (0.33, 0.48)
Other nuts	X-25524			**0.27**	**1.13 × 10^−11^**	0.87	**0.27**	**3.75 × 10^−12^**	0.81	0.41 (0.33, 0.48)
	X-25523			**0.26**	**1.41 × 10^−11^**	0.86	**0.26**	**3.57 × 10^−11^**	0.81	0.49 (0.42, 0.56)
	X-23970			**0.27**	**2.17 × 10^−12^**	0.86	**0.25**	**2.48 × 10^−10^**	0.79	0.52 (0.46, 0.59)
Seeds	X-11847			0.15	2.06 × 10^−4^	0.75	**0.26**	**1.63 × 10^−11^**	0.76	0.60 (0.54, 0.66)
	X-11858			0.13	7.09 × 10^−4^	0.74	**0.24**	**5.89 × 10^−10^**	0.76	0.50 (0.43, 0.56)
	X-18059			**0.24**	**1.10 × 10^−9^**	0.74	0.18	3.92 × 10^−6^	0.71	0.32 (0.24, 0.40)
**DAIRY/DAIRY ALTERNATIVES**										
Milk	phenylacetylglycine	Peptide	Acetylated Peptides	**0.40**	**2.79 × 10^−26^**	0.87	**0.28**	**4.49 × 10^−13^**	0.79	0.53 (0.47, 0.59)
	2,8-quinolinediol sulfate	Xenobiotics	Food Component/Plant	**0.31**	**3.17 × 10^−15^**	0.84	0.19	1.24 × 10^−6^	0.77	0.50 (0.43, 0.57)
	*N,N,N*-trimethyl-5-aminovalerate	Amino Acid	Lysine Metabolism	**0.30**	**1.02 × 10^−14^**	0.83	**0.28**	**1.67 × 10^−12^**	0.79	0.40 (0.33, 0.48)
Almond milk or rice milk	*N,N,N*-trimethyl-5-aminovalerate	Amino Acid	Lysine Metabolism	−0.16	5.33 × 10^−5^	0.71	**−0.21**	**1.38 × 10^−7^**	0.69	0.40 (0.33, 0.48)
	catechol sulfate	Xenobiotics	Benzoate Metabolism	**0.22**	**1.69 × 10^−8^**	0.71	**0.22**	**1.20 × 10^−8^**	0.65	0.71 (0.66, 0.75)
	X-25800			0.16	3.56 × 10^−5^	0.69	**0.21**	**1.20 × 10^−7^**	0.65	0.33 (0.26, 0.42)
Total cheese	heptenedioate (C7:1-DC) *	Lipid	Fatty Acid, Dicarboxylate	**0.24**	**2.07 × 10^−9^**	0.88	**0.22**	**2.14 × 10^−8^**	0.78	0.49 (0.42, 0.55)
	4-methylhexanoylglutamine	Lipid	Fatty Acid Metabolism (Acyl Glutamine)	**0.24**	**1.87 × 10^−9^**	0.87	**0.23**	**5.23 × 10^−9^**	0.78	0.51 (0.44, 0.57)
	glutamine conjugate of C9H16O2 (1) *	Partially Characterized Molecules	Partially Characterized Molecules	0.15	2.23 × 10^−4^	0.86	**0.22**	**1.26 × 10^−8^**	0.78	0.52 (0.46, 0.59)
Cream	glucuronide of C_19_H_28_O_4_ (1)*	Partially Characterized Molecules	Partially Characterized Molecules	**0.39**	**4.89 × 10^−24^**	0.80	0.13	1.22 × 10^−3^	0.70	0.83 (0.79, 0.85)
	X-25500			**0.34**	**4.09 × 10^−19^**	0.79	0.12	1.80 × 10^−3^	0.68	0.63 (0.57, 0.68)
	X-12738			**0.35**	**8.62 × 10^−20^**	0.78	0.10	1.22 × 10^−2^	0.68	0.72 (0.67, 0.76)
**FATS AND OILS**										
Creamy salad dressing	carnitine of C_10_H_14_O_2_ (2) *	Partially Characterized Molecules	Partially Characterized Molecules	**0.26**	**4.09 × 10^−11^**	0.78	0.16	5.02 × 10^−5^	0.67	0.39 (0.32, 0.47)
	X-24363			**0.26**	**4.26 × 10^−11^**	0.77	0.18	4.13 × 10^−6^	0.67	0.56 (0.50, 0.62)
	X-13693			**0.22**	**1.04 × 10^−8^**	0.77	0.19	1.81 × 10^−6^	0.67	0.47 (0.40, 0.54)
Oil and vinegar salad dressing	*N*-methyltaurine	Amino Acid	Methionine, Cysteine, SAM and Taurine Metabolism	0.16	5.45 × 10^−5^	0.76	**0.20**	**2.63 × 10^−7^**	0.80	0.51 (0.44, 0.57)
Olive oil	*N*-methyltaurine	Amino Acid	Methionine, Cysteine, SAM and Taurine Metabolism	**0.24**	**4.33 × 10^−10^**	0.80	0.20	7.40 × 10^−7^	0.76	0.51 (0.44, 0.57)
	X-25419			**0.24**	**4.25 × 10^−10^**	0.79	0.18	5.28 × 10^−6^	0.74	0.55 (0.49, 0.61)
	X-17733			**−0.21**	**6.36 × 10^−8^**	0.78	−0.13	7.26 × 10^−4^	0.74	0.50 (0.44, 0.57)
**MISCELLANEOUS**										
French fries	pentose acid *	Partially Characterized Molecules	Partially Characterized Molecules	**−0.27**	**2.28 × 10^−12^**	0.85	−0.07	1.00 × 10^−1^	0.72	0.57 (0.50, 0.62)
	abscisate	Xenobiotics	Food Component/Plant	**−0.28**	**6.40 × 10^−13^**	0.85	−0.03	4.65 × 10^−1^	0.72	0.47 (0.40, 0.54)
	catechol sulfate	Xenobiotics	Benzoate Metabolism	**−0.24**	**1.01 × 10^−9^**	0.84	−0.09	2.68 × 10^−2^	0.72	0.71 (0.66, 0.75)
Chips	glutamine conjugate of C8H12O2 (2) *	Partially Characterized Molecules	Partially Characterized Molecules	**0.25**	**9.16 × 10^−11^**	0.80	**0.25**	**1.45 × 10^−10^**	0.76	0.45 (0.38, 0.52)
	glucuronide of C_10_H_14_O_2_ (1) *	Partially Characterized Molecules	Partially Characterized Molecules	**0.28**	**6.97 × 10^−13^**	0.80	0.18	8.55 × 10^−6^	0.74	0.42 (0.35, 0.50)
	X-23970			**−0.20**	**2.30 × 10^−7^**	0.79	−0.10	1.45 × 10^−2^	0.72	0.52 (0.46, 0.59)
Chocolate candy	X-12823			**0.38**	**4.94 × 10^−23^**	0.85	**0.30**	**3.18 × 10^−14^**	0.83	0.40 (0.33, 0.48)
	3-methylxanthine	Xenobiotics	Xanthine Metabolism	**0.32**	**1.98 × 10^−16^**	0.83	**0.27**	**4.96 × 10^−12^**	0.82	0.60 (0.54, 0.66)
	7-methylurate	Xenobiotics	Xanthine Metabolism	**0.32**	**1.50 × 10^−16^**	0.83	**0.28**	**3.94 × 10^−13^**	0.82	0.62 (0.56, 0.67)
Dark chocolate	theobromine	Xenobiotics	Xanthine Metabolism	**0.29**	**6.72 × 10^−14^**	0.79	**0.23**	**3.38 × 10^−9^**	0.71	0.58 (0.52, 0.64)
	X-12823			**0.32**	**5.05 × 10^−17^**	0.79	**0.25**	**1.36 × 10^−10^**	0.69	0.40 (0.33, 0.48)
	3, 7-dimethylurate	Xenobiotics	Xanthine Metabolism	**0.30**	**2.03 × 10^−14^**	0.79	**0.22**	**1.76 × 10^−8^**	0.69	0.56 (0.50, 0.62)
Desserts	X-24340			**0.21**	**8.12 × 10^−8^**	0.80	0.15	1.96 × 10^−4^	0.74	0.50 (0.44, 0.57)
	3, 4-methylene heptanoylglycine	Lipid	Fatty Acid Metabolism (Acyl Glycine)	**0.21**	**1.32 × 10^−7^**	0.80	0.10	1.07 × 10^−2^	0.73	0.56 (0.50, 0.62)
Bars	X-16649			**0.21**	**5.23 × 10^−8^**	0.81	0.20	4.42 × 10^−7^	0.73	0.53 (0.46, 0.59)
	2-(4-hydroxyphenyl)propionate	Xenobiotics	Benzoate Metabolism	**0.21**	**1.13 × 10^−7^**	0.80	0.15	1.13 × 10^−4^	0.71	0.36 (0.29, 0.44)
	sucralose	Xenobiotics	Food Component/Plant	**0.21**	**1.41 × 10^−7^**	0.80	0.11	5.11 × 10^−3^	0.70	0.50 (0.43, 0.56)
Soy sauce	X-11847			0.17	9.43 × 10^−6^	0.74	**0.22**	**2.46 × 10^−8^**	0.67	0.60 (0.54, 0.66)
	X-11849			0.18	2.84 × 10^−6^	0.74	**0.20**	**2.50 × 10^−7^**	0.66	0.63 (0.58, 0.69)
Artificial sweeteners	sucralose	Xenobiotics	Food Component/Plant	**0.31**	**5.88 × 10^−16^**	0.75	**0.35**	**2.71 × 10^−19^**	0.77	0.50 (0.43, 0.56)
	acesulfame	Xenobiotics	Food Component/Plant	**0.21**	**4.72 × 10^−8^**	0.75	**0.25**	**2.78 × 10^−10^**	0.73	0.49 (0.43, 0.56)
	X-25785			**0.23**	**3.71 × 10^−9^**	0.72	0.17	1.59 × 10^−5^	0.68	0.48 (0.41, 0.55)
**ALCOHOL**										
Total alcohol	ethyl glucuronide	Xenobiotics	Chemical	**0.65**	**5.84 × 10^−78^**	0.99	**0.59**	**1.08 × 10^−60^**	0.94	0.63 (0.57, 0.68)
	ethyl alpha-glucopyranoside	Xenobiotics	Food Component/Plant	**0.57**	**4.01 × 10^−56^**	0.97	**0.48**	**2.15 × 10^−38^**	0.90	0.53 (0.46, 0.59)
	2,3-dihydroxyisovalerate	Xenobiotics	Food Component/Plant	**0.44**	**5.03 × 10^−31^**	0.92	**0.42**	**1.22 × 10^−28^**	0.86	0.46 (0.39, 0.53)
Beer	ethyl glucuronide	Xenobiotics	Chemical	**0.45**	**5.34 × 10^−33^**	0.89	**0.41**	**5.84 × 10^−27^**	0.86	0.63 (0.57, 0.68)
	ethyl alpha-glucopyranoside	Xenobiotics	Food Component/Plant	**0.43**	**4.82 × 10^−30^**	0.86	**0.38**	**9.29 × 10^−24^**	0.84	0.53 (0.46, 0.59)
	2,3-dihydroxy-3-methylvalerate	Amino Acid	Leucine, Isoleucine and Valine Metabolism	**0.30**	**2.87 × 10^−14^**	0.83	**0.28**	**7.16 × 10^−13^**	0.82	0.42 (0.35, 0.50)
Total wine	ethyl glucuronide	Xenobiotics	Chemical	**0.62**	**1.86 × 10^−68^**	0.94	**0.49**	**7.76 × 10^−39^**	0.84	0.63 (0.57, 0.68)
	ethyl alpha-glucopyranoside	Xenobiotics	Food Component/Plant	**0.51**	**1.55 × 10^−43^**	0.91	**0.39**	**7.03 × 10^−24^**	0.78	0.53 (0.46, 0.59)
	X-17306			**0.52**	**1.81 × 10^−45^**	0.89	**0.48**	**5.30 × 10^−38^**	0.83	0.57 (0.51, 0.63)
Red wine	ethyl glucuronide	Xenobiotics	Chemical	**0.54**	**3.62 × 10^−50^**	0.88	**0.41**	**1.48 × 10^−27^**	0.80	0.63 (0.57, 0.68)
	ethyl alpha-glucopyranoside	Xenobiotics	Food Component/Plant	**0.45**	**1.47 × 10^−33^**	0.84	**0.32**	**2.12 × 10^−16^**	0.76	0.53 (0.46, 0.59)
	2,3-dihydroxy-3-methylvalerate	Amino Acid	Leucine, Isoleucine and Valine Metabolism	**0.44**	**7.17 × 10^−32^**	0.81	**0.36**	**2.02 × 10^−20^**	0.77	0.42 (0.35, 0.50)
White wine	ethyl glucuronide	Xenobiotics	Chemical	**0.44**	**4.89 × 10^−31^**	0.78	**0.36**	**2.24 × 10^−21^**	0.73	0.63 (0.57, 0.68)
	ethyl alpha-glucopyranoside	Xenobiotics	Food Component/Plant	**0.36**	**1.22 × 10^−20^**	0.75	**0.31**	**1.76 × 10^−15^**	0.71	0.53 (0.46, 0.59)
	X-17306			**0.35**	**1.62 × 10^−19^**	0.74	**0.36**	**6.39 × 10^−21^**	0.72	0.57 (0.51, 0.63)
Liquor	ethyl glucuronide	Xenobiotics	Chemical	**0.42**	**1.65 × 10^−28^**	0.79	**0.25**	**1.26 × 10^−10^**	0.70	0.63 (0.57, 0.68)
	ethyl alpha-glucopyranoside	Xenobiotics	Food Component/Plant	**0.36**	**2.17 × 10^−20^**	0.77	0.19	2.12 × 10^−6^	0.67	0.53 (0.46, 0.59)
	*N*-acetyltaurine	Amino Acid	Methionine, Cysteine, SAM and Taurine Metabolism	**0.27**	**7.24 × 10^−12^**	0.73	0.16	7.99 × 10^−5^	0.68	0.63 (0.58, 0.69)
**BEVERAGES**										
Total coffee	glucuronide of C_19_H_28_O_4_ (1) *	Partially Characterized Molecules	Partially Characterized Molecules	**0.83**	**0.56 × 10^165^**	1.00	**0.81**	**0.82 × 10^145^**	0.99	0.83 (0.79, 0.85)
	citraconate/glutaconate	Energy	TCA Cycle	**0.71**	**0.35 × 10^100^**	1.00	**0.69**	**2.65 × 10^−89^**	0.97	0.77 (0.73, 0.81)
	feruloylquinate (3)	Xenobiotics	Food Component/Plant	**0.68**	**2.40 × 10^−86^**	0.99	**0.66**	**4.46 × 10^−79^**	0.97	0.71 (0.67, 0.76)
Decaffeinated	glucuronide of C_19_H_28_O_4_ (1) *	Partially Characterized Molecules	Partially Characterized Molecules	**0.24**	**1.83 × 10^−9^**	0.66	0.20	4.26 × 10^−7^	0.63	0.83 (0.79, 0.85)
	quinate	Xenobiotics	Food Component/Plant	**0.21**	**1.36 × 10^−7^**	0.65	0.16	4.67 × 10^−5^	0.63	0.82 (0.79, 0.85)
	X-25666			**0.21**	**9.93 × 10^−8^**	0.65	0.18	5.43 × 10^−6^	0.63	0.72 (0.67, 0.76)
Caffeinated	glucuronide of C_19_H_28_O_4_ (1) *	Partially Characterized Molecules	Partially Characterized Molecules	**0.78**	**0.27 × 10^129^**	0.98	**0.76**	**0.68 × 10^121^**	0.97	0.83 (0.79, 0.85)
	3-hydroxypyridine glucuronide	Xenobiotics	Chemical	**0.69**	**2.27 × 10^−91^**	0.98	**0.66**	**2.06 × 10^−80^**	0.95	0.77 (0.73, 0.80)
	3-hydroxypyridine	Xenobiotics	Chemical	**0.72**	**0.71 × 10^101^**	0.98	**0.67**	**1.20 × 10^−84^**	0.95	0.76 (0.72, 0.80)
Total tea	*N*-acetyltheanine	Xenobiotics	Food Component/Plant	**0.52**	**9.90 × 10^−46^**	0.93	**0.51**	**1.04 × 10^−43^**	0.88	0.55 (0.48, 0.61)
	coumaroylquinate (1)	Xenobiotics	Food Component/Plant	**0.36**	**6.16 × 10^−21^**	0.83	**0.35**	**1.02 × 10^−19^**	0.79	0.53 (0.47, 0.60)
	2-methoxyresorcinol sulfate	Xenobiotics	Chemical	**0.31**	**9.47 × 10^−16^**	0.81	**0.33**	**1.20 × 10^−17^**	0.76	0.62 (0.56, 0.67)
Green tea	*N*-acetyltheanine	Xenobiotics	Food Component/Plant	**0.29**	**6.14 × 10^−14^**	0.74	**0.34**	**7.48 × 10^−19^**	0.72	0.55 (0.48, 0.61)
	*S*-adenosylhomocysteine (SAH)	Amino Acid	Methionine, Cysteine, SAM and Taurine Metabolism	**−0.26**	**4.23 × 10^−11^**	0.73	**−0.26**	**4.40 × 10^−11^**	0.66	0.42 (0.35, 0.50)
	X-12740			**0.25**	**7.96 × 10^−11^**	0.72	**0.22**	**2.62 × 10^−8^**	0.66	0.41 (0.33, 0.48)
Black tea	*N*-acetyltheanine	Xenobiotics	Food Component/Plant	**0.41**	**1.17 × 10^−27^**	0.81	**0.47**	**4.63 × 10^−36^**	0.81	0.55 (0.48, 0.61)
	2-methoxyresorcinol sulfate	Xenobiotics	Chemical	**0.25**	**8.05 × 10^−11^**	0.73	**0.27**	**5.46 × 10^−12^**	0.72	0.62 (0.56, 0.67)
	1,2,3-benzenetriol sulfate (2)	Xenobiotics	Chemical	**0.25**	**1.98 × 10^−10^**	0.73	**0.26**	**4.95 × 10^−11^**	0.71	0.57 (0.51, 0.63)
Herbal tea	X-12306			**0.24**	**1.75 × 10^−9^**	0.74	0.19	1.77 × 10^−6^	0.65	0.66 (0.61, 0.71)
	X-23423			**0.22**	**2.43 × 10^−8^**	0.74	0.15	1.18 × 10^−4^	0.63	0.51 (0.45, 0.58)
	catechol sulfate	Xenobiotics	Benzoate Metabolism	**0.22**	**3.74 × 10^−8^**	0.73	0.16	7.34 × 10^−5^	0.63	0.71 (0.66, 0.75)
Sugar-sweetened beverages	X-23970			**−0.22**	**3.64 × 10^−8^**	0.82	−0.12	2.96 × 10^−3^	0.77	0.52 (0.46, 0.59)
	X-23424			**0.20**	**2.10 × 10^−7^**	0.81	0.05	2.27 × 10^−1^	0.76	0.28 (0.20, 0.37)
	hydroxy-N6, N6, N6-trimethyllysine *	Amino Acid	Lysine Metabolism	**0.24**	**4.34 × 10^−10^**	0.81	0.08	3.37 × 10^−2^	0.76	0.62 (0.56, 0.67)
Diet beverages	X-25785			**0.47**	**4.73 × 10^−36^**	0.84	**0.43**	**1.04 × 10^−29^**	0.81	0.48 (0.41, 0.55)
	acesulfame	Xenobiotics	Food Component/Plant	**0.42**	**1.45 × 10^−28^**	0.83	**0.32**	**1.15 × 10^−16^**	0.77	0.49 (0.43, 0.56)
	sucralose	Xenobiotics	Food Component/Plant	**0.41**	**1.79 × 10^−27^**	0.81	**0.28**	**7.01 × 10^−13^**	0.74	0.50 (0.43, 0.56)

^1^. Diet–metabolite correlations in **bold** had *p* < 3.56 × 10^−7^ for FFQ and *p* < 3.42 × 10^−7^ for average 24 h diet recalls and |*r*| > 0.2 from Pearson’s partial correlation analysis. Adjusted for age, gender, race/ethnicity, education, smoking status, physical activity, body mass index, ethanol consumption (except for alcohol-containing items), and energy intake. CPS-3, Cancer Prevention Study-3; DAS, Diet Assessment Sub-study. ^2^. Biochemical name of metabolite correlated with respective food or food group. Metabolites starting with X are unnamed and the super pathway of these is unknown. Asterisk (*) represents putative identity that has not been officially confirmed based on a standard. (1) and (2) indicate that the metabolite differs from another with the same mass in the position of the R group. CMPF, 3-carboxy-4-methyl-5-propyl-2-furanpropanoate. ^3^. ICC, intraclass correlation coefficient, to assess the reproducibility of the identified food-related metabolites over six months. ^4^. Items are only available on 24 h-diet recalls.

## Data Availability

Data described in the manuscript and analytic code are not available to protect participant confidentiality and in adherence with institutional policies.

## Supplementary Materials

The following are available online at https://www.mdpi.com/article/10.3390/metabo11040248/s1. Figure S1: study population exclusion; Table S1: Food–metabolite associations identified using either FFQ or 24 h diet recalls in the CPS-3 Diet Assessment Sub-study; Table S2: Food–metabolite associations identified using the FFQ in the CPS-3 Diet Assessment Sub-study; Table S3: Food–metabolite associations identified using the average 24 h diet recalls in the CPS-3 Diet Assessment Sub-study; Table S4: Food group definitions in the CPS-3 Diet Assessment Sub-study.
