# Supplementary figures and images for: Identification and Reproducibility of Urinary Metabolomic Biomarkers of Habitual Food Intake in a Cross-Sectional Analysis of the Cancer Prevention Study-3 Diet Assessment Sub-Study
Source: Metabolites. 2021 Apr 17;11(4):248. doi: 10.3390/metabo11040248 (PMC8072637; doi:10.3390/metabo11040248)

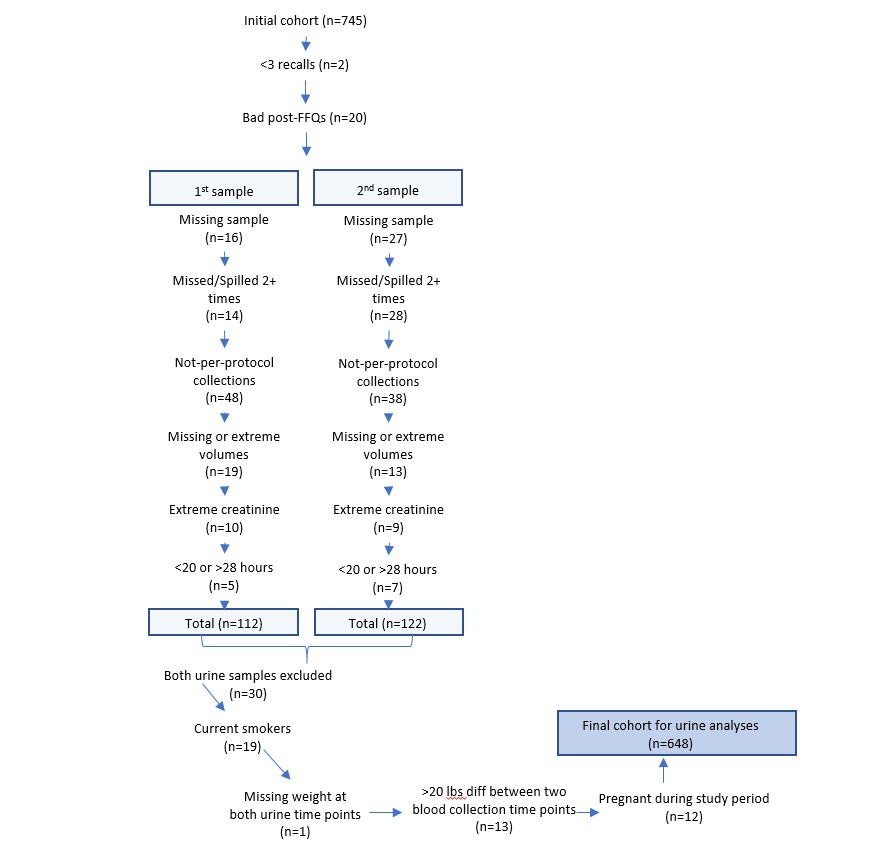

Supplement: Supplementary file 1 [file metabolites-11-00248-s001.zip › metabolites-1025256-supplementary/Supplementary Figure1.jpg]
